# Information Theoretic Causality Detection between Financial and Sentiment Data

**DOI:** 10.3390/e23050621

**Published:** 2021-05-16

**Authors:** Roberta Scaramozzino, Paola Cerchiello, Tomaso Aste

**Affiliations:** 1 Department of Economics and Management, University of Pavia, Via San Felice 7, 27100 Pavia, Italy; paola.cerchiello@unipv.it; 2 Department of Computer Science, University College London, Gower Street, London WC1E 6EA, UK; t.aste@ucl.ac.uk; 3 UCL Centre for Blockchain Technologies, University College London, London WC1E 6BT, UK; 4 Systemic Risk Centre, London School of Economics and Political Sciences, London WC2A 2AE, UK

**Keywords:** information theory, textual analysis, transfer entropy, financial news, causality, time series

## Abstract

The interaction between the flow of sentiment expressed on blogs and media and the dynamics of the stock market prices are analyzed through an information-theoretic measure, the transfer entropy, to quantify causality relations. We analyzed daily stock price and daily social media sentiment for the top 50 companies in the Standard & Poor (S&P) index during the period from November 2018 to November 2020. We also analyzed news mentioning these companies during the same period. We found that there is a causal flux of information that links those companies. The largest fraction of significant causal links is between prices and between sentiments, but there is also significant causal information which goes both ways from sentiment to prices and from prices to sentiment. We observe that the strongest causal signal between sentiment and prices is associated with the Tech sector.

## 1. Introduction

Causality is hard to detect from observations. This is because the occurrence of two events, one after the other, does not necessarily imply that the first caused the second. In 1969, Granger [1] first proposed to look at causality in terms of the amount of extra information that the observation of a variable provides about another variable. In its original formulation, this corresponds to an additional term in a linear regression for financial forecasting, but the idea is general and requires the quantification of information flow between variables.

In finance, the relationships between companies are usually analyzed considering the so-called “hard” information such as stock prices, trade volumes, the quantity of output, but, in recent years, there has been an increase in the use of “soft” information including textual data, opinions, news, and sentiment. Indeed, the economic value of things and firms is both material and immaterial. Reputation is playing a major role in economics. This has probably always been true, but it has become even more crucial in the present world where social-media has a pervasive role. Therefore, a current study of market behaviour cannot be limited to the hard evidence related to the financial metrics but must also dig into the soft metrics of social media and news. The relation between the two is still a domain in exploration.

On the one hand, an efficient market hypothesis would suggest that all information must be comprised into the prices. On the other hand, swings in social opinions have their independent dynamics and sometimes follow and other times anticipate market movements. In this paper, we further investigate such relationship by means of information theoretic tools, with the aim of understanding the manifest and latent dynamics of hard and soft information within the US market.

We analyze the causality between some of the most important worldwide companies using both hard (prices) and soft (social media sentiment) information and investigate their interrelations. Causality is quantified through tools of information theory using entropy and mutual information. The first represents the uncertainty related to a variable’s possible outcomes and quantifies its information content, the second one measures the information that two variables share. The transfer entropy is a conditional mutual information between the past of a variable and the future of another variable conditioned to the past of this second variable. It measures the information transferred between the two variables or equivalently the reduction in uncertainty uniquely caused by a variable on the another [2].

### 1.1. Background: Textual Analysis in Finance

The use of textual analysis in the financial sector is relatively recent but constantly growing. Among the earlier papers, Engelberg [3] demonstrates that soft information, although more difficult to calculate, offers greater predictability on asset prices in particular at a longer horizon. Tirea and Negru [4] create an optimized portfolio through the combination of text mining, sentiment analysis, and risk models on the Bucharest Stock Exchange. Jothimani et al. [5] in their study integrate hard and soft data, the latter collected from online articles and tweets, and demonstrate that the combination of the two types of information allows optimization of the investment portfolio. Zheludev et al. [6] using sentiment techniques on social media messages show that, analyzing the S&P index, information contained in social media can impact financial market forecasts. The authors [7] use the content of regular financial news to track the evolution across time and space of topics which are relevant in the financial context.

With a focus on the impact of negative sentiment, Tetlock [8], using daily content from the Wall Street journal, finds that the volume of market exchanges is determined by unusually high or low pessimistic values. Indeed, Huang et al. [9] show that investors react differently depending on whether the information received is positive or negative; in the latter case, the reaction is stronger. They also find, on a non-market-based test, evidence that information extracted from analyst reports has predictive power on earnings growth over the following five years.

Due to the easier processing of short text data, a notable application of sentiment analysis in finance has involved the analysis of tweets. Bollen et al. [10] examine whether the collective mood (based on six social moods: Calm, Alert, Sure, Vital, Kind, and Happy), obtained from all the tweets published in a given period in the USA, is correlated or predictive of DJIA (Dow Jones Industrial Average) values. They observe that only some of the six moods are correlated with DJIA values, with a lag of 3–4 days. Zhang et al. [11] find that, by analyzing the sentiment spikes on Twitter posts, it is possible to predict what will happen in the market the following day. Rao et al. [12] using Granger’s Causality Analysis show that, in the short term, tweets influence the trend in stock prices; Ranco et al. [13] considering 30 joint-stock companies of the DJIA index, through the “study of events” methodology [14], a technique used in economics and finance that analyzes abnormal price changes linked to external events; for each stock, it highlights the external events grouped according to a measure of polarity. They relate the prevailing sentiment in financial tweets, in terms of volume, and stock returns showing a statistically significant dependence. Souza et al. [15] studying retail brands analyze if there is a significant connection between sentiment and volume of tweets with volatility and return on stock prices, seeing that the data obtained from social media are relevant to understand the financial dynamics and, in particular, demonstrate how the sentiment obtained from the tweets is linked to the returns more than traditional news-wires.

You and Luo [16] investigate classification accuracy using textual and visual data. Carvalho et al. [17] classify tweets through an approach where paradigm words are selected using a genetic algorithm.

Kolchyna et al. [18] describe different techniques for classification of Twitter messages: lexicon based method and machine learning method, and present a new method that combines the two techniques. The score obtained from the lexicon based method is the input feature for the machine learning approach, and they demonstrate that classifications are more accurate using this combined technique.

In the field of financial risk management, Cerchiello and Giudici [19] construct a systemic risk model with a combination of financial tweets and financial prices to comprehensively assess the impact of systemic risk.

### 1.2. Background: Information Theory

Information theory was born in 1948 with the publication of Claude Shannon’s article [20]. It stands at the interface of several multidisciplinary fields of research such as: mathematics, statistics, physics, telecommunications, and computer science, and it is applied to various fields, including the financial one.

Particularly used in the financial field is the concept of entropy. Dimpfl and Peter [21], analyzing through entropy the flow of information between CDS (Credit default swap) and the bond market, show that information flows in both directions with the importance of the CDS market increasing over time. Kwon and Yang [22], using entropy, examine the flow of information between composite stock indices and individual stocks and show that this flow is stronger from indices to stocks than vice versa. Shreiber [23] theorizes the concept of transfer entropy as a measure of oriented coherence statistics between systems that evolve over time and Marschinski and Kants [24], following this concept, analyze the flow of information between two time series: Dow Jones and DAX stock index. They introduce a modified estimator able to perform well also in the case of short temporal series. Baek et al. [25] analyze, in the US stock market, the strength and direction of information using Transfer Entropy and conclude that companies in the energy and electricity sector influence the entire market. Nicola et al. [26] analyze the US banking network, made up of the top 74 listed banks, with the aim of highlighting whether mutual information and transfer entropy are able to Granger causing financial stress indices and the USD/CHF exchange rate. For the implementation of the analysis, they used general and partial Granger causality, the latter correlated to representative measures of the general economic condition.

The main goal, in the present work, is to investigate the causal relationship between two events. We chose the asymmetric information-theoretic measure identified as transfer entropy, to detect strength and direction of transfer information between sentiment and prices. Differently from Granger Causality, we use a nonlinear estimation of the transfer entropy.

The design of the paper is organized as follows: Section 2 presents the methodology used, Section 3 presents a description of the data, in Section 4, we report the results, and conclusions are presented in Section 5.

## 2. Methods

In our work, we use a nonlinear transfer entropy estimation, first introduced in [23], to identify and quantify causality between time series.

Using Shannon’s measure of information [20], we can denote the uncertainty associated with a variable *X* by:

(1) $$H\left(X\right)=-\sum_{x}p\left(x\right)\log_{2}p\left(x\right);$$

This quantity can be conditioned on a second variable to obtain conditional entropy:

(2) H(X|Y)=H(X,Y)−H(Y);

while the information that *X* and *Y* share is instead the so-called mutual information:

(3) I(X,Y)=H(Y)−H(Y|X)=H(X)−H(X|Y);

It expresses how the knowledge of a variable reduces the uncertainty of another, and it is symmetric in *X* and *Y*.

We can express the information transfer from *X* to *Y* in terms of conditional mutual information for a given lag *k*:

(4) $$TE_{\left(X\to Y\right)}^{\left(k\right)}=I\left(Y_{t},X_{t-k}|Y_{t-k}\right)=H\left(Y_{t}|Y_{t-k}\right)-H\left(Y_{t}|X_{t-k},Y_{t-k}\right);$$

Equation (4) quantifies the amount of uncertainty on $Y_{t}$ reduced by the knowledge of the lagged variable $X_{t-k}$ given the information of the lagged variable $Y_{t-k}$ itself. It is therefore a quantification of the additional information on variable *Y* provided by the past of variable *X* taking into account what is already known about the past of *Y*.

This expression is general and applies to either linear and nonlinear estimations. In the linear case, one uses multivariate normal modeling, in the nonlinear case, one can instead estimate Transfer Entropy with a non-parametric density estimation that directly uses the empirical frequencies of observations into histogram bins.

In this paper, following [27], we adopt such a non-parametric, nonlinear approach and estimate the joint entropy using the multidimensional histogram tool available from the ‘PyCausality’ Python package (https://github.com/ZacKeskin/PyCausality (accessed on 15 May 2021). According to such method, the observation space is divided into bins and the observations are allocated to each bin depending on their value. It is evident that the appropriate choice of bins is crucial. We chose the equi-probable bins approach, which enforces that, in each bin, the number of data points is approximately the same. In previous studies [27], it was shown that this approach yields the best results for artificial data where the true underlying causality structure is known. In our case, where the causality structure must be discovered, we verified that other choices, such as equi-sized bins, return similar results on our dataset; however, the equi-probable bins provide the cleanest outputs.

A limitation of this non-parametric approach is that it requires a large number of observations. Indeed, for the transfer entropy between two variables, we have to estimate a three-dimensional histogram. In general, for *p* variables, the dimension is at least d=p+1. For any meaningful statistical analysis, the bins in the histogram must be populated and therefore one must have a number of observations that is larger than (number of bins${)}^{d}$. This method is non-parametric; however, the choice of the number of bins is important, and this could be seen as a hyper-parameter. In the present study, however, the choice is highly constrained by the sample size. We have indeed two years of observations (512 days, see Section 3). Therefore, the maximum number of bins should be no larger than ${\left(512\right)}^{1/3}=8$. In [27], it was shown that results are robust for a range of different values of the number of bins. Indeed, we tested the bin number in a range between 3 and 8 obtaining consistently similar results. We eventually decided for a number of bins equal to 5, which was giving the cleanest result. It should be clear that, with this non-parametric approach, with the present dataset, it would be unfeasible to extend the analysis to greater dimensions beyond the computation of transfer entropies between two variables.

Another important choice is the lag *k*. We chose the first-order lag k=1, since we assume that one day of delay is enough to see the effects of a variable on another. This is because, in an increasingly connected world, news spread almost immediately around the world. Similarly, the time for one event to impact another is extremely close. As robustness check, we have also tested a higher number of lags up to 5, obtaining consistent results with the one here reported for k=1.

The transfer entropy returns a non-negative real value. The greater the number, the larger is the amount of information measured. However, there is no reference and the number itself, without a benchmark, is of little interest. In order to obtain such a reference, we compared it with a null-hypothesis from data sets where any causal relation is removed. Such data were obtained from the original ones by shuffling randomly the time sequence of observations. In this way, we obtained both a null-hypothesis reference and its statistics. From the mean $\langle {TE}_{shuffle}\rangle$ and the standard deviation $\sigma_{shuffle}$ of the shuffled transfer entropy, we computed the statistical significance of the Transfer entropy results in terms of the following *Z*-score:

(5) $$Z:=\frac{TE-\langle {TE}_{shuffle}\rangle }{\sigma_{shuffle}}.$$

The *Z*-score provides a distance, measured in terms of standard deviations, of the observed transfer entropy with respect to expected value for non-causally related variables. Larger *Z*-scores imply a value of the transfer entropy that is more significantly deviating from the values expected when the variables are not causally related, implying therefore a larger likelihood of causal relation. In this paper, we used 50 shuffles. We use the *Z*-score because it is a robust statistical validation that depends on minimal assumptions. We checked the quantiles as well, retrieving consistent results. However, with only 50 shuffles, the quantile measure tends to be noisier. We shuffle single entries only; therefore, we eliminate autocorrelations. Shuffling blocks instead could have produced noisier null-hypothesis transfer entropy potentially yielding to slightly lower Z scores.

Finally, we made use of the *Z*-score to construct graphs of significant causal links by retaining causality links at different threshold values, namely Z>2 and Z>3. On the resulting networks, the community detection algorithm were applied to identify causality structures. We also compared the networks between themselves and with respect to a reference network based on news.

For a better understanding of the employed methodology, hereafter we describe the step by step analysis workflow.


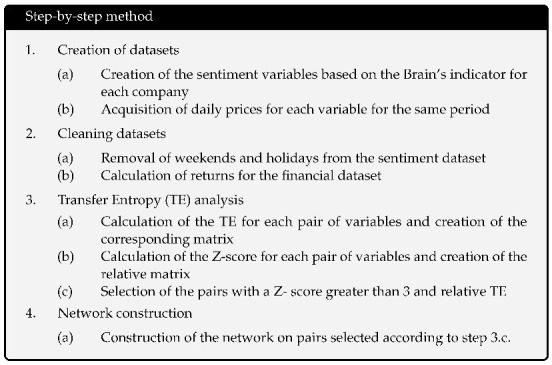


## 3. Data

In this paper, we consider the top 50 companies of S&P. The complete list of companies with the corresponding ticker code and rank Capitalization is available in Table 1.

We analyze two different types of information: stock prices and sentiment index.

The sentiment index is provided by Brain (link to the site: https://braincompany.co/ (accessed on 15 May 2021). For each day, in a period starting from November 2018 to November 2020, a sentiment value is calculated from news and blogs written in English for each and every company. A brain sentiment indicator is represented by a value ranging between −1 to 1, where −1 corresponds to a negative sentiment, 0 to a neutral sentiment, and + 1 to a positive sentiment.

The workflow of Brain Sentiment indicator is described in the box.


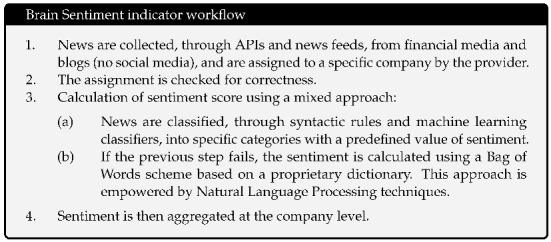


For the same period, we have daily stock prices for each company from Yahoo Finance. Since the sentiment index is available every day, differently from market data, we exclude weekend days with regards to the former, in order to have comparable time series.

For the daily stock prices, we calculate the logarithmic return

(6) $$L=\log\left(Price_{t}\right)-\log\left(Price_{t-1}\right),$$

which is a rate of change of the variable. We apply such transformation just to financial data because the sentiment index is already a stable variable in a range between −1 and 1. We performed the Anderson–Darling test and verified that all sentiment variables can be considered stationary with null-hypothesis *p*-values all below 5%. We perform stationary tests on log returns too, and the results are the same as for sentiment variables. In Appendix B, we add two plots for the time series (the first for returns and the second for sentiment) and also two more images on a subset for an improved visualization. This result could be deemed as a bit surprising in the light of the COVID-19 virus outbreak started in the spring of 2020, but, as already showed in [28], such shock had a small impact on the overall statistics of sentiment time series.

After these pre-processing steps, we obtain a complete dataset, with values on the same scale for a total of 100 variables (50 prices log-returns and 50 sentiment index) and 515 observations (two years of work-daily data).

## 4. Results

As explained in the previous sections, we want to assess the possible causal relationship between stock price and sentiment indicator focusing on some of the largest worldwide companies. To this end, we compute the transfer entropy and the relative *Z*-score for all couples of variables (market price and sentiment index). We have therefore 100 variables and 100×99=9900 distinct couples.

**Figure 1:**
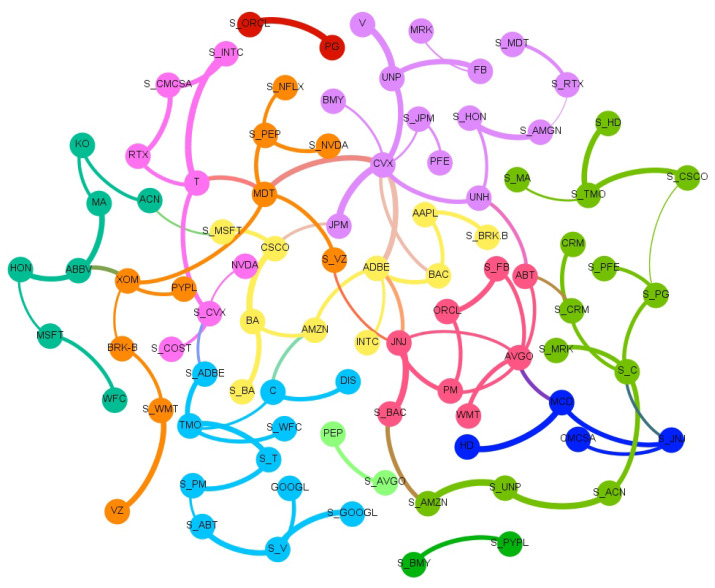
Network of links with Z score larger than 3. The colors represent the 12 Communities found using a Community detection algorithm. The sentiment index timeseries is indicated with an S before the ticker’s name. The clockwise direction of the curves indicates the direction of connections. Moreover, the reader can notice that there are not bidirectional interactions (to and from one vertex) and there are no cycles (paths that can be run across starting and ending with the same vertex) except for AVGO, JNJ, and PM. This result is not an imposed constraint of the algorithm but rather a result of the analysis.

The full network of causality links without imposing any restriction is too dense. The large number of links and the significant density of the graph prevent inferring useful and insightful information. A more detailed and consistent analysis is depicted in Figure 1, where a sub-network which retains only causal links with *Z*-scores larger than 3 is shown. Such a stringent score allows for the presence of the most significant links. Figure 1 clearly zooms in on a fraction of the connections easing the readability. In this figure, and in all others, the clockwise direction of the arcs between nodes indicates the direction of connections. Note that, despite the fact that we estimated the transfer entropy between couples of variables, from the network in Figure 1, we can also infer properties for the relations between a higher number of variables and assess the presence of potential confounding factors. Indeed, any larger multivariate causality structure will reveal itself as a clique in the graph and any confounding factor will form a cycle. We observe only one clique of dimension three with a directional cycle JNJ → AVGO → PM → JNJ.

For a more comprehensive understanding and readability, we report in Table 2 and Table 3 the associated Transfer Entropy values and the *Z*-score for each couple of stock with a *Z*-score larger than 3.

The three tables report results classified according to the S&P industry sectors: Consumer discretionary, Consumer staples, Energy, Healthcare, Tech, Financial, Industrial and Communications. The sectors are not homogeneously populated, in particular, Healthcare and Tech ones have the largest number of stocks, respectively, 10 and 15 companies. Whilst the sector’s classification is important for the correct assessment of the pattern drivers, the tendency of big companies to diversify the types of business more and more is unquestionable. As an example, Amazon, which is listed in the Consumer discretionary sector, has a division named ‘Amazon Web Services’ for cloud computing and device and a division named ‘Amazon Studios’ for music and videos streaming. Bear in mind that the division among the sectors does not completely reflect the real connections among the companies.

A Community Detection algorithm [29] is employed to investigate the presence of meaningful communities inside our network in Figure 1.

The community detection algorithm implemented is the Louvain method [30], a heuristic method that is based on modularity optimization. It is an unsupervised algorithm that partitions the network into mutually exclusive communities in two steps: modularity optimization with local node relocation and community aggregation. We selected this algorithm due to its simplicity and computational efficiency.

The community algorithm finds 12 different communities as we can see from the different colors. Most of the communities are similar in terms of number of companies. Interestingly, such groups have some recognizable overlap with S&P sectors, but also distinctive features revealing the different nature of market price and sentiment interconnections, which goes well beyond companies’ core business.

By looking at the connections in such a network, we can distinguish between variables associated with the price returns (identified generically as ‘price’ hereafter) and variables associated instead with sentiment scores (identified generically as ‘sentiment’ hereafter).

We observe that most of the links are from Price to Price (See Table 2), followed by the links from Sentiment to Sentiment (see Table 3) and then the Sentiment to Price and finally Price to Sentiment (see Table 4). We observe an interesting asymmetry between companies and sectors that are influencers and the others that are followers with most of the significant links involving two different industry sectors. The leading one, in terms of number of significant links, is the Technological sector with a predominance of connection towards the Consumer sector: Accenture causing (→) Coca-Cola; Mastercard → Coca-Cola; Broadcom → Philip Morris; Oracle → Philip Morris; Amazon → Adobe; McDonald’s → Broadcom; Walmart → Broadcom. The influence is also very interesting of different sectors onto the Energy one: Bank of America, Bristol, JPMorgan, Medtronic, UnitedHealth and Union Pacific cause Chevron; while Paypal causes Exxon. We note that this abundance of links to the energy sector is unique to this Price to Price network. There are also several links within the same sectors: a connection between United health → Abbot, both in the Healthcare sector; McDonald’s → Home Depot, in the Consumer sector; and Adobe → Intel in the Tech sector.

There are also numerous links in the Sentiment to Sentiment network (see in Table 3). In this case, many links are related to the Healthcare sector, most of them are relationships between the Healthcare and the Consumer sector: Johnson&Johnson → Walt Disney; Merck&Co → Walt Disney; Thermo Fisher → Home Depot; Pfizer → Procter&Gabmble; and Abbott → Philip Morris. We also find links between companies in the same sector: Pepsi → Netflix; and Walt Disney → Procter&Gamble.

In the Price to Sentiment network (Table 4), we notice that there is a significant frequency of stocks related to the Healthcare sector which affect other sectors: Tech (Thermo Fisher → Adobe, Abbott → Salesforce.com); Financial (Johnson&Johnson → Bank of America); Consumer (Medtronic → Pepsi); and Communications (Thermo Fisher → AT&T, Johnson&johnson → Verizon and Medtronic → Verizon).

Perhaps the most interesting result lays upon the causal links from Sentiment to Price (Table 4). Most of them are in the Technological sector in particular Tech to Tech: Microsoft → Accenture; Facebook → Broadcom; Salesforce.com, Microsoft → Cisco; and Facebook → Oracle.

The analysis reveals a dominant role of Healthcare and Technology both as influencer and follower sectors across all four networks. Another important sector is Consumer, both essential (staples) and discretionary, which are, however, mainly followers and less influencers.

**Figure 2:**
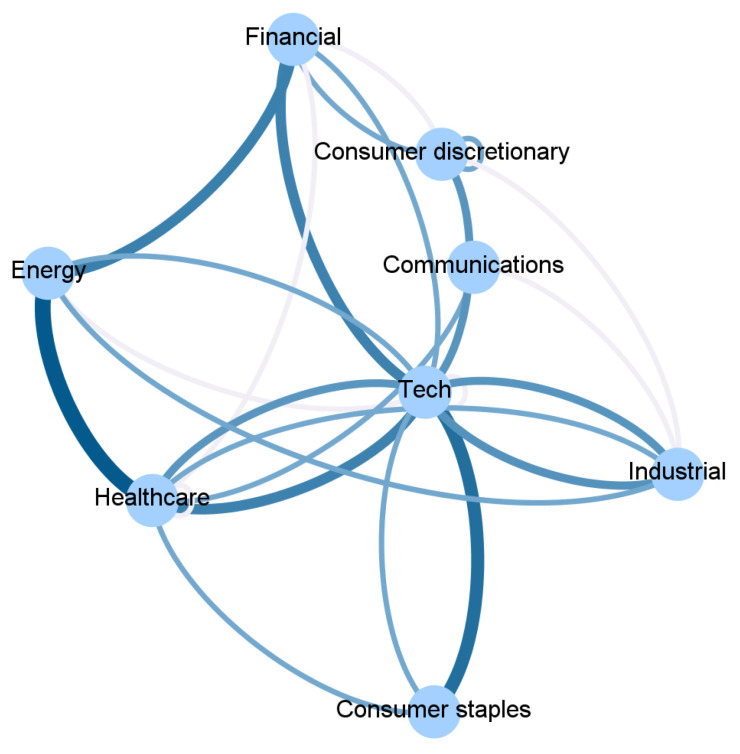
The aggregated Price → Price network visualization of Table 2, Table 3 and Table 4 representing the flows of influence among sectors quantified as total, significant (Z > 3), transfer entropy exchanged in each direction. The clockwise direction of the curves indicates the direction of connections.

**Figure 3:**
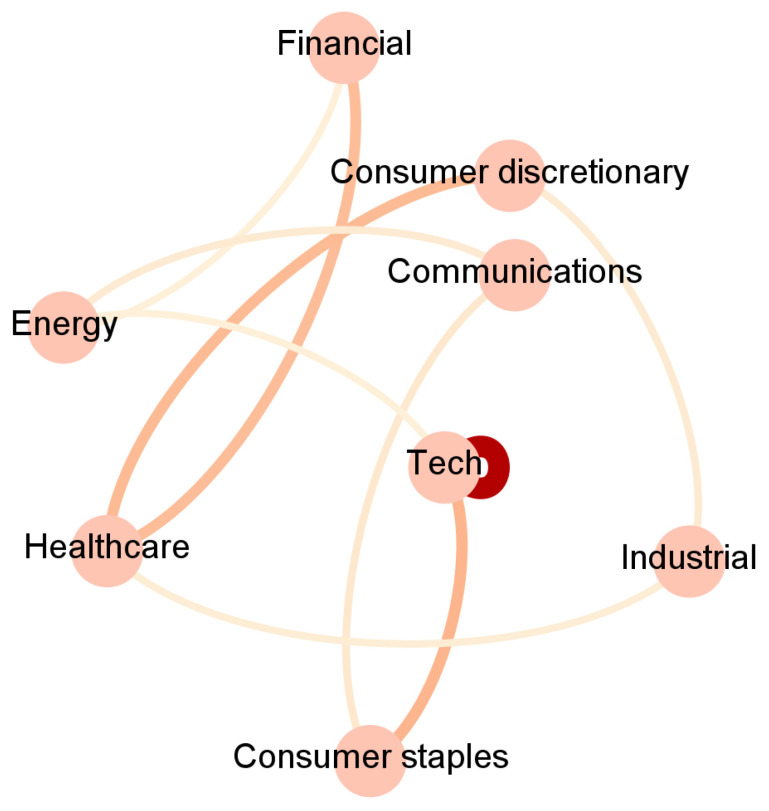
The aggregated Sentiment → Price network visualization of Table 2, Table 3 and Table 4 representing the flows of influence among sectors quantified as total, significant (Z > 3), transfer entropy exchanged in each direction. The clockwise direction of the curves indicates the direction of connections.

**Figure 4:**
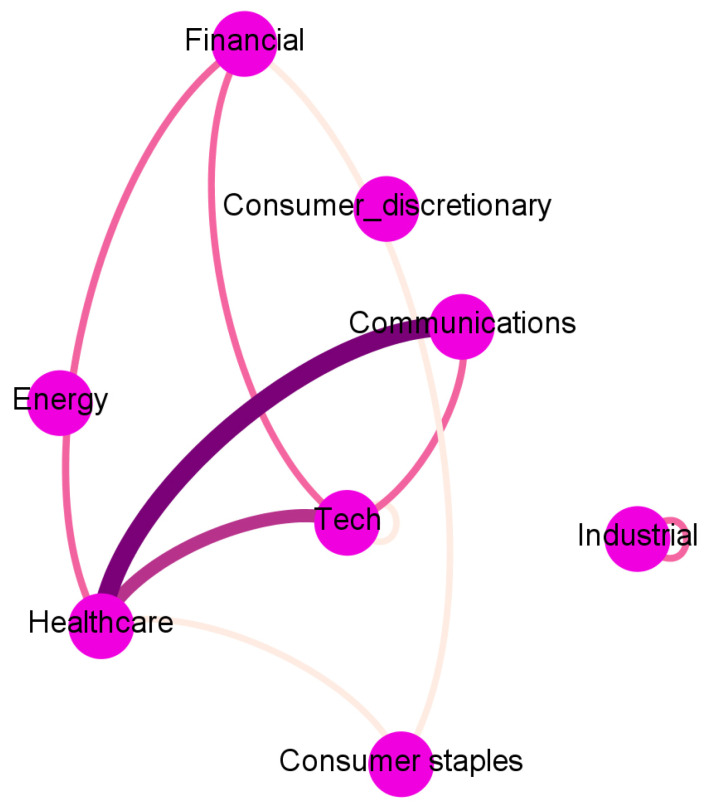
The aggregated Price → Sentiment network visualization of Table 2, Table 3 and Table 4 representing the flows of influence among sectors quantified as total, significant (Z > 3), transfer entropy exchanged in each direction. The clockwise direction of the curves indicates the direction of connections.

**Figure 5:**
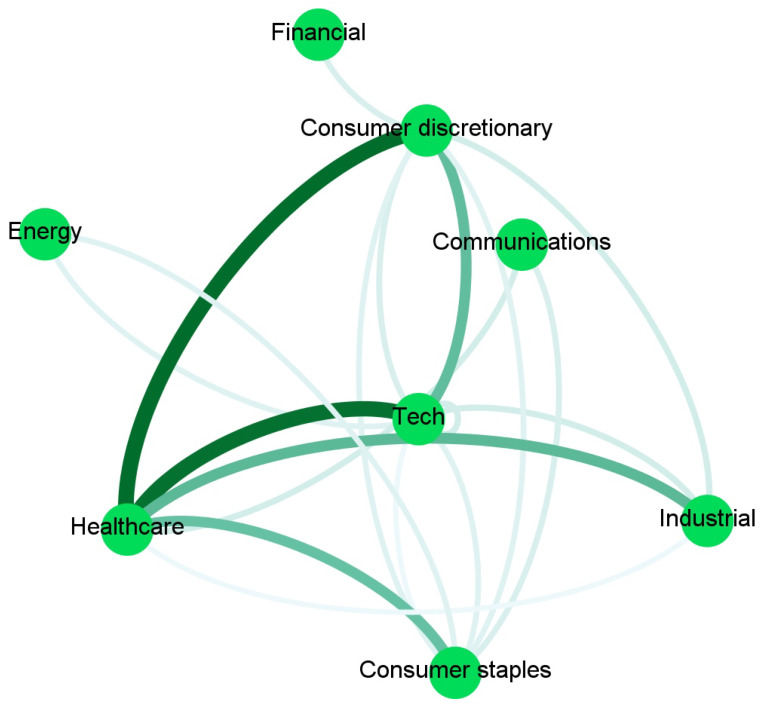
The aggregated Sentiment → Sentiment network visualization of Table 2, Table 3 and Table 4 representing the flows of influence among sectors quantified as total, significant (Z > 3), transfer entropy exchanged in each direction. The clockwise direction of the curves indicates the direction of connections.

To ease the interpretation, we report in Figure 2, Figure 3, Figure 4 and Figure 5 and Appendix A an aggregated network visualization of Table 2, Table 3 and Table 4 representing the flows of influence between industry sectors quantified as total, significant (Z>3), transfer entropy exchanged in each direction. This analysis allows for a global view of the eight sectors in terms of reciprocal influence. We note that the four networks have very distinct characteristics.

Specifically, in the Price→Price network in Figure 2, we observe a role of the energy sector, being a follower of both Financial and Healthcare sectors, a role that is not revealed in any of the other networks. Moreover, we stress that the financial sector, which traditionally plays a pivotal role when the financial market is considered, appears to be not so predominant. Indeed, the largest average Transfer Entropy is measured from Healthcare to Energy with 0.92. These results are in line with [28], which showed that the healthcare sector increased the level of importance (expressed in terms of network connectivity) during the waves of the pandemic outbreak in the US market.

The Sentiment→Price network in Figure 3 has a major self-influencing loop with the sentiment on the Technological sector affecting its own price (TE 0.92); it also reveals some influence of the Financial sector on Healthcare (TE 0.36) and Healthcare on Consumer Discretionary (TE 0.37).

In the Price→Sentiment network in Figure 4, the main leading role is played by Healthcare, and the role of the Communication sector as a follower of Healthcare (TE 0.55) and as an influencer of Technology (TE 0.2) also emerges. This is not present in any of the other networks. Healthcare is also influencing Technology (TE 0.37).

Finally, the Sentiment→Sentiment network in Figure 5 shows a dominating role of Healthcare that is affecting the Consumer sectors (TE 0.56), Industry (TE 0.38), and Technology (TE 0.55).

Overall, the Price→Price network has the largest number of connections i.e., 25, then Sentiment→Sentiment follows with 19, finally Sentiment→Price and Pirce→Sentiment with, respectively, 10 and 9.

### Comparison between TE Matrix and Dataset Based on News

Since one of the main aims of our paper is to disentangle the role played by the information disclosed through news and measured by means of a sentiment score, we further analyze such component. To deepen our investigation, we pay greater attention to the sentiment aspect carrying out a further analysis using data concerning news provided by the Brain (link to the site: https://braincompany.co/, accessed on 15 May 2021) to identify relations between stocks by counting the number of times two tickers are mentioned within the same news article.

**Figure 6:**
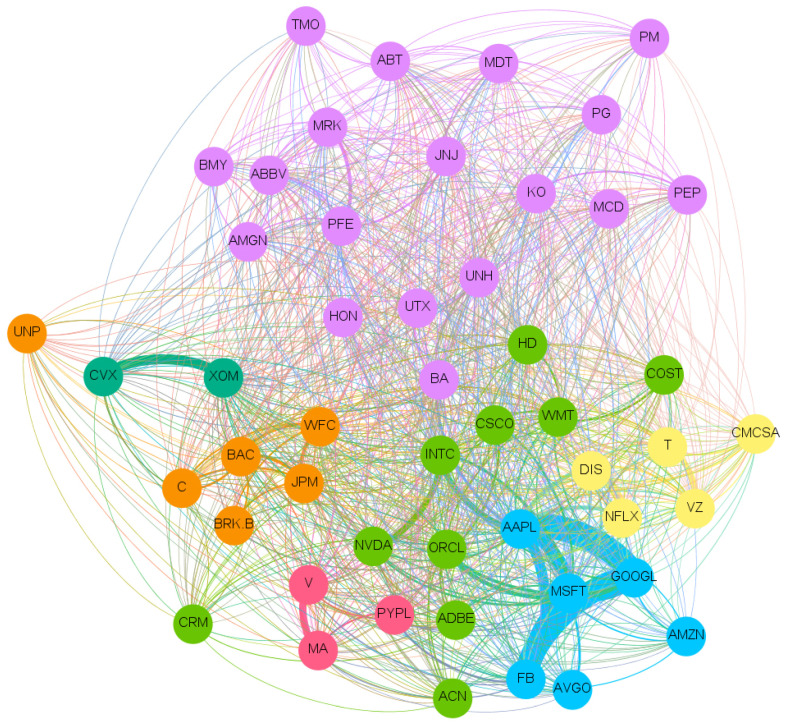
Network news in common. The colours represent the seven communities found using a Community detection algorithm. The clockwise direction of the curves indicates the direction of connections.

In Figure 6, we report the complete network of news in common. As already happened with unrestricted analysis, the network appears to be too dense to be readable. However, some clear patterns are already evident, like the strict connections among the company giants like AAPL, MSFT, GOOGL, FB, and AMZN (bottom right in blue), which indeed represent a community per se.

**Figure 7:**
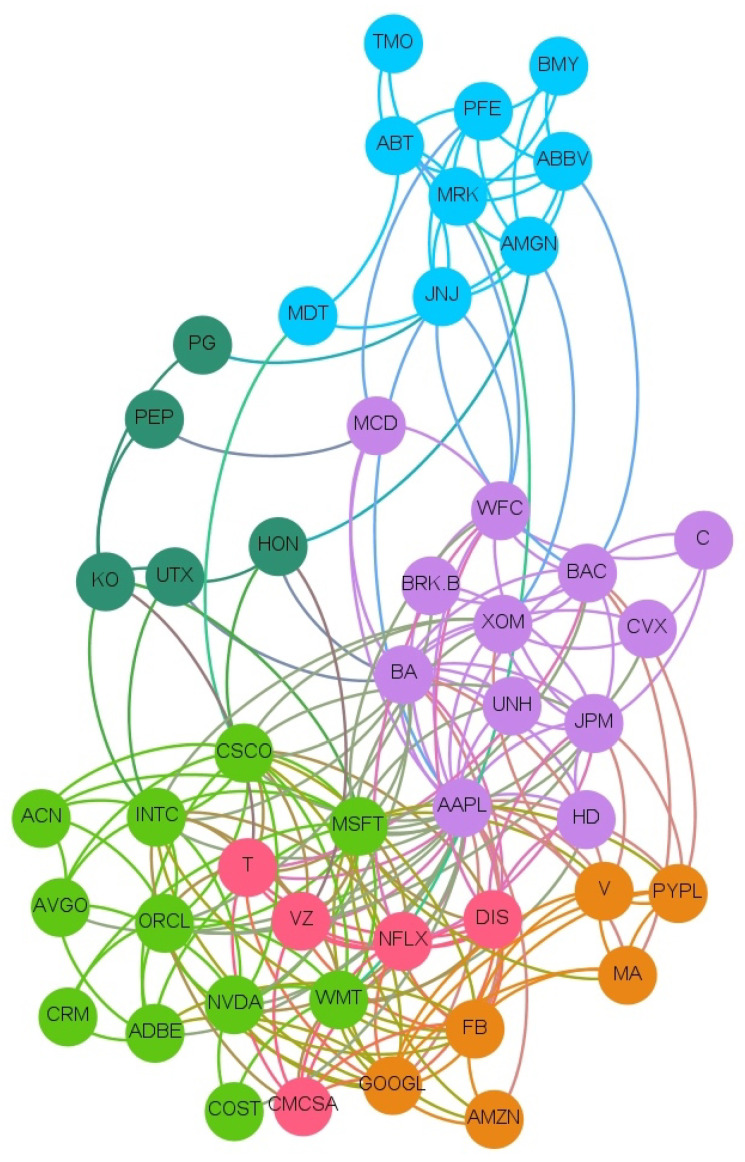
Network news in common larger than 20. The colours represent the seven communities found using a community detection algorithm. The clockwise direction of the curves indicates the direction of connections.

To ease the readability, we filter out the less significant links; thus, in Figure 7, we report the network built by retaining only the connections between stocks that score a number of news in common larger than a threshold value of 20 (such value has been identified after some sensitivity analysis).

Such a network is then compared with the previous causality networks for Price to Price (PP) Figure 2, Sentiment to Price (SP) Figure 3, Price to Sentiment (PS) Figure 4, and Sentiment to Sentiment (SS) Figure 5 obtained by imposing on the links a threshold Z-score value.

Results for the thresholds: Z>2.5 and a number of news in common larger than 20 are reported in Table 5. The reader can see that there is a rather modest overlap between the networks that mostly involve very popular companies.

In order to statistically quantify the significance of such overlap between the networks, we compute the hypergeometric probability to have a certain number or more of overlapping edges in two directed graphs. Of course, results depend upon the chosen thresholding for the number of news and the Z-score. Overall, we find that there is no statistical significance in terms of *p*-value for the thresholds Z>2.5 and News > 20. However, this does not mean that the links are just by chance.

By performing a sensitivity analysis by changing the threshold values, we observe that the four networks have different patterns. The Price to Price causality network shows relations with news with a rather large number of overlaps and statistical significance with *p*-values below 1% but only when the network is less restricted using a small news threshold and small Z-scores. This seems to indicate that news pick some insights of the internal dynamics of the market and that identify correctly important events in the financial domain that trigger propagation of information through the social media. This significance at small thresholds could indicate that this happens on average, but the importance of the news or the intensity of the causality relation is not relevant.

For what concerns the other networks, we observe that larger thresholds (more restrictive condition and less links) for the number of news in common increase statistical significance. This could indicate that news are identifying events that also resonate on the social media, but this tend to happen only for events with high relevance.

## 5. Discussion and Conclusions

In this paper, we study the causal relationships between opinions reflected on blogs and media and the patterns in stock market values, in order to investigate causal interactions between these variables. We focus on top 50 companies of the S&P index rooted in different sectors: Consumer discretionary, Consumer staples, Energy, Healthcare, Tech, and Financial Industrial and Communications. Data cover two years from November 2018 through November 2020. In our analysis, we employ an information-theoretic measure, the transfer entropy, to monitor the information flows between sentiment and market movements. We use a recently developed nonlinear methodology [27] that can better capture causality extending the traditional Granger approach.

Our information-theoretic analysis revealed a large number of strong connections. As expected, the highest number of significant causal relationships between companies involves the same kind of data source (price → price, sentiment → sentiment), but there are also strong connections across different data sources.

Some sectors are more influential in terms of sentiment dynamics and less in terms of price dynamics. For instance, in the sentiment to sentiment network, we can clearly spot the pivotal role of the Healthcare sector which influences both the consumer discretionary and the technological sectors. Such pattern is present, although with differentiated importance within the other networks too. What is surprising is the role of the Financial sector, which is traditionally in a paramount position compared to other sectors. Our analysis shows that financial companies are still important if we restrict to price data solely or if we consider the impact of sentiment on price but much less within the alternative scenarios. However, this is in line with what was already reported in [31] where a reduction of centrality of the financial sector was pointed out. This was also reported by [28], where, through a temporal dynamic network analysis, the authors show that the financial sector behaves differently as an isolated cluster which reacts mainly to market price data (more on such peculiar pattern in [32]). Another important sector is the technological one, either as influencer or follower depending on the network we may consider. The remaining sectors seem less consistent and change in relevance and role across the different networks.

From this study, we can conclude, first of all, that mutual influences between various companies are not limited to influences between companies within the same sector. On the contrary, the cross sector interactions tend to be more relevant. This might be because companies with high capitalization tend to operate in many markets other than their core business. Secondly, the price variables show a more homogeneous behavior, with connections which tend to be stronger and also more frequent. Nonetheless, we identify several cases where sentiment about a company has a strong influence on sentiment on other companies and also to other company prices. In particular, the Tech sector reveals a very strong influence of sentiment on prices. This might be a consequence of the presence of the most popular companies in terms of branding, the ‘Big Five’ (Google, Amazon, Facebook, Microsoft and Apple), which are often mentioned in news and blogs and this continuous notoriety obviously affects the financial aspect. The present paper can be improved and extended into several directions: US companies should be complemented and compared with European ones which typically show different patterns and level of connectedness.

## Figures and Tables

**Table 1:** Detailed description of the top 50 S&P companies.

Rank	Stock	Ticker
Communication		
13	AT & T Inc.	T
18	Verizon Comm. Inc.	VZ
Consumer Discretionary		
3	Amazon.com Inc.	AMZN
26	Comcast Corp.	CMCSA
14	Walt Disney Co.	DIS
19	Home Depot Inc.	HD
34	McDonald’s Corp.	MCD
37	Netflix Inc.	NFLX
Consumer Staples		
39	Costco Wholesale Corp.	COST
24	Coca-Cola Co.	KO
28	PepsiCo Inc.	PEP
10	Procter & Gamble Co.	PG
43	Philip Morris Int. Inc.	PM
30	Walmart Inc.	WMT
Financial		
12	Bank of America Corp	BAC
5	Berkshire Hathaway Inc.	BRK.B
29	Citigroup Inc.	C
6	JPMorgan Chase & Co.	JPM
22	Wells Fargo & Co.	WFC
Industrial		
25	Boeing Co.	BA
42	Honeywell Int. Inc.	HON
47	Union Pacific Corp.	UNP
50	Raytheon Technologies	RTX
Healthcare		
41	AbbVie Inc.	ABBV
31	Abbott Laboratories	ABT
36	Amgen Inc.	AMGN
38	Bristol-Myers Squibb Co.	BMY
8	Johnson & Johnson	JNJ
33	Medtronic Plc	MDT
20	Merck & Co. Inc.	MRK
23	Pfizer Inc.	PFE
46	Thermo Fisher Scientific Inc.	TMO
15	UnitedHealth Group Inc.	UNH
Tech		
2	Apple Inc.	AAPL
44	Accenture Plc	ACN
32	Adobe Inc.	ADBE
45	Broadcom Inc.	AVGO
35	Salesforce.com Inc.	CRM
27	Cisco Systems Inc.	CSCO
4	Facebook Inc.	FB
7	Alphabet Inc.	GOOGL
16	Intel Corp.	INTC
17	Mastercard Inc.	MA
1	Microsoft Corp.	MSFT
40	NVIDIA Corp.	NVDA
49	Oracle Corp.	ORCL
48	PayPal Holdings Inc.	PYPL
9	Visa Inc.	V
Energy		
21	Chevron Corp.	CVX
11	Exxon Mobil Corp.	XOM

**Table 2:** Couples of stocks with relative transfer Entropy, $TE_{\left(X\to Y\right)}^{\left(1\right)}$, values, Z scores larger than 3 (in brackets), and sectors for Price to Price network. The sectors are indicated with the capital letter; in particular, we have F for Financial, H for Healthcare, T for Tech, I for Industrial, CD for Consumer discretionary, CS for Consumer staples, C for Communications, and E for Energy.

Var X	Var Y	Value TE (Zscore)	Sectors
Price to Price			
T	MDT	0.18 (4.24)	C→H
MSFT	WFC	0.18 (4.24)	T→F
PM	JNJ	0.18 (3.99)	CS→H
T	RTX	0.18 (3.98)	C→I
V	UNP	0.20 (3.98)	T→I
ABBV	HON	0.19 (3.81)	H→I
MCD	HD	0.19 (3.80)	CD→CD
MDT	CVX	0.19 (3.76)	H→E
UNP	FB	0.19 (3.75)	I→T
MSFT	HON	0.17 (3.66)	T→I
WMT	AVGO	0.18 (3.64)	CS→T
BAC	ADBE	0.18 (3.64)	F→T
JPM	CVX	0.20 (3.63)	F→E
UNP	CVX	0.19 (3.61)	I→E
ABBV	XOM	0.18 (3.54)	H→E
DIS	C	0.18 (3.38)	CD→F
MA	ABBV	0.19 (3.3)	T→H
C	AMZN	0.18 (3.36)	F→CD
AVGO	PM	0.1 (3.35)	T→CS
BA	CSCO	0.2 (3.35)	I→T
AAPL	BAC	0.18 (3.34)	T→F
UNH	ABT	0.18 (3.33)	H→H
CVX	ADBE	0.19 (3.33)	E→T
BRK-B	XOM	0.17 (3.26)	F→E
ORCL	PM	0.18 (3.24)	T→CS
MA	KO	0.18 (3.24)	T→CS
ADBE	INTC	0.18 (3.24)	T→T
BAC	CVX	0.18 (3.22)	F→E
ADBE	JNJ	0.18 (3.22)	T→H
C	TMO	0.18 (3.16)	F→H
FB	MRK	0.17 (3.15)	T→H
AMZN	BA	0.18 (3.13)	CD→I
MDT	XOM	0.18 (3.11)	H→E
BMY	CVX	0.17 (3.11)	H→E
PYPL	XOM	0.18 (3.10)	T→E
CSCO	JPM	0.18 (3.1)	T→F
UNH	CVX	0.19 (3.06)	H→E
ABT	AVGO	0.18 (3.05)	H→T
ACN	KO	0.18 (3.04)	T→CS
JNJ	AVGO	0.18 (3.04)	H→T
AMZN	ADBE	0.18 (3.02)	CD→T
MCD	AVGO	0.18 (3.00)	CD→T

**Table 3:** Couples of stocks with relative transfer Entropy, $TE_{\left(X\to Y\right)}^{\left(1\right)}$, values, Z scores larger than 3 (in brackets) and sectors for Sentiment to Sentiment networks. The sectors are indicated with the capital letter; in particular, we have F for Financial, H for Healthcare, T for Tech, I for Industrial, CD for Consumer discretionary, CS for Consumer staples, C for communications, and E for Energy.

Var X	Var Y	Value TE (Zscore)	Sectors
Sentiment to Sentiment			
AMGN	HON	0.2 (4.63)	H→I
AMZN	UNP	0.2 (4.57)	CD→I
C	CRM	0.18 (4.51)	F→T
C	ACN	0.19 (4.47)	F→T
TMO	CSCO	0.19 (4.36)	H→T
AMZN	BAC	0.19 (4.34)	CD→F
BMY	PYPL	0.19 (4.3)	H→T
TMO	HD	0.2 (4.04)	H→CD
V	ABT	0.2 (3.97)	T→H
V	GOOGL	0.2 (3.89)	T→T
INTC	CMCSA	0.19 (3.82)	T→CD
ACN	UNP	0.2 (3.79)	T→I
NVDA	PEP	0.18 (3.57)	T→CS
MRK	C	0.19 (3.45)	H→F
T	PM	0.19 (3.42)	C→CS
PFE	PG	0.18 (3.33)	H→CS
ABT	PM	0.17 (3.32)	H→CS
TMO	MA	0.17 (3.32)	H→T
C	PG	0.18 (3.31)	F→CS
MDT	RTX	0.18 (3.12)	H→I
CVX	COST	0.18 (3.09)	E→CS
PEP	NFLX	0.18 (3.08)	CS→CD
JNJ	C	0.18 (3.07)	H→F
ADBE	CVX	0.18 (3.07)	T→E
RTX	AMGN	0.16 (3.04)	I→H
PG	CSCO	0.16 (3.03)	CS→T

**Table 4:** Couples of stocks with relative transfer Entropy, $TE_{\left(X\to Y\right)}^{\left(1\right)}$, values, Z scores larger than 3 (in brackets) and sectors for Price to Sentiment, and Sentiment to Price networks. The sectors are indicated with the capital letter; in particular, we have F for Financial, H for Healthcare, T for Tech, I for Industrial, CD for Consumer discretionary, CS for Consumer staples, C for communications, and E for Energy.

Var X	Var Y	Value TE (Zscore)	Sectors
Price to Sentiment			
JNJ	BAC	0.2 (4.37)	H→F
TMO	ADBE	0.19 (4.05)	H→T
TMO	T	0.19 (3.92)	H→C
T	INTC	0.20 (3.83)	C→ T
ABT	CRM	0.18 (3.54)	H→T
BA	BA	0.19 (3.51)	I→I
MDT	VZ	0.18 (3.36)	H→C
AAPL	BRK.B	0.18 (3.34)	T→F
BRK-B	WMT	0.18 (3.18)	F→CS
JNJ	VZ	0.17 (3.08)	H→C
GOOGL	V	0.18 (3.06)	T→T
MDT	PEP	0.18 (3.05)	H→CS
Sentiment to Price			
CVX	T	0.19 (4.34)	E→C
ORCL	PG	0.20 (4.24)	T→CS
FB	ORCL	0.19 (4.01)	T→T
WMT	VZ	0.12 (3.83)	CS→C
WFC	TMO	0.18 (3.68)	F→H
MSFT	ACN	0.17 (3.64)	T→T
CMCSA	RTX	0.19 (3.61)	CD→I
JNJ	CMCSA	0.18 (3.41)	H→CD
AVGO	PEP	0.18 (3.38)	T→CS
JNJ	MCD	0.19 (3.37)	H→CD
JPM	PFE	0.17 (3.29)	F→H
HON	UNH	0.18 (3.19)	I→H
CVX	NVDA	0.17 (3.17)	E→T
MSFT	CSCO	0.19 (3.12)	T→T
JPM	CVX	0.17 (3.06)	F→E
CRM	CRM	0.19 (3.06)	T→T
FB	AVGO	0.18 (3.03)	T→T

**Table 5:** Overlap between links in news network and links in Transfer Entropy matrix with a threshold on news equal to 20 and on Z-score equal to 2.5.

var_x	var_y
Price to Price variables (PP)	
NVDA	BA
BAC	AAPL
CMCSA	T
CSCO	BA
CSCO	NVDA
CSCO	ORCL
HD	JPM
INTC	T
JPM	CSCO
BA	NVDA
NVDA	MSFT
PYPL	JPM
PYPL	MSFT
WFC	MSFT
Price to Sentiment variables (PS)	
JNJ	BAC
ABBV	BMY
AAPL	BRK.B
BAC	BRKB.B
T	INTC
GOOGL	V
CSCO	WMT
Sentiment to Price variables (SP)	
MSFT	AAPL
MSFT	ACN
MSFT	CSCO
MSFT	GOOGL
CRM	ORCL
MSFT	PYPL
ABT	TMO
Sentiment to Sentiment variables (SS)	
FB	ADBE
INTC	CMCSA
ADBE	CRM
INTC	CSCO
V	GOOGL
AMGN	HON
FB	V
XOM	MSFT

**Figure A1:**
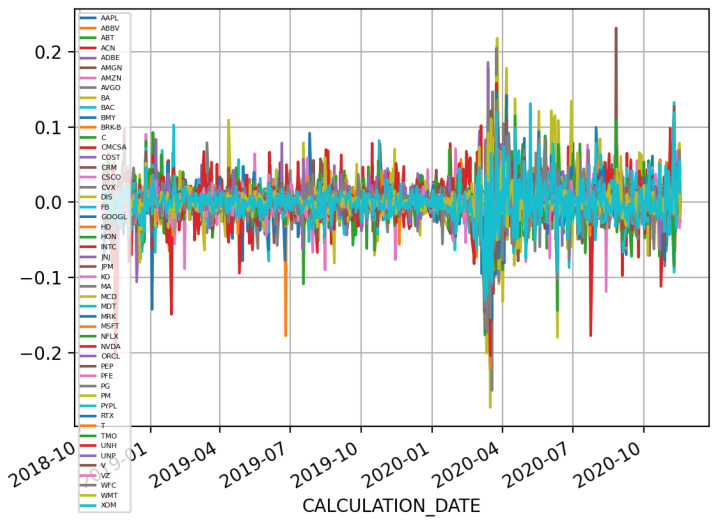
Time series plot of price variables (returns).

**Figure A2:**
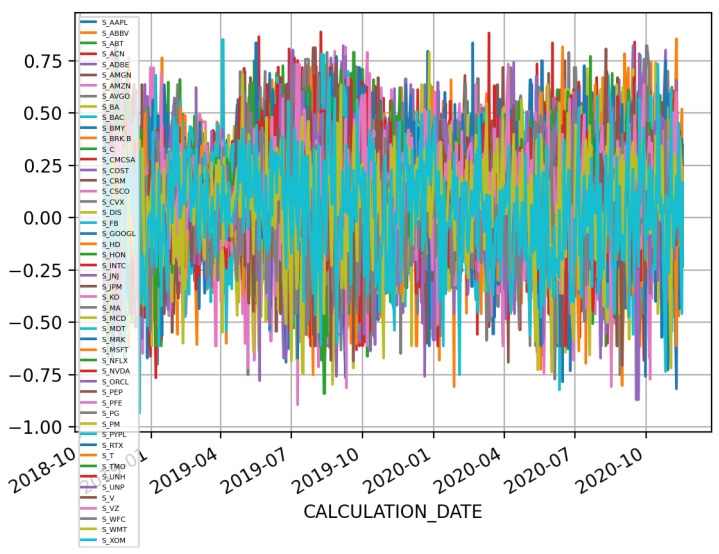
Time series plot of sentiment variables.

**Figure A3:**
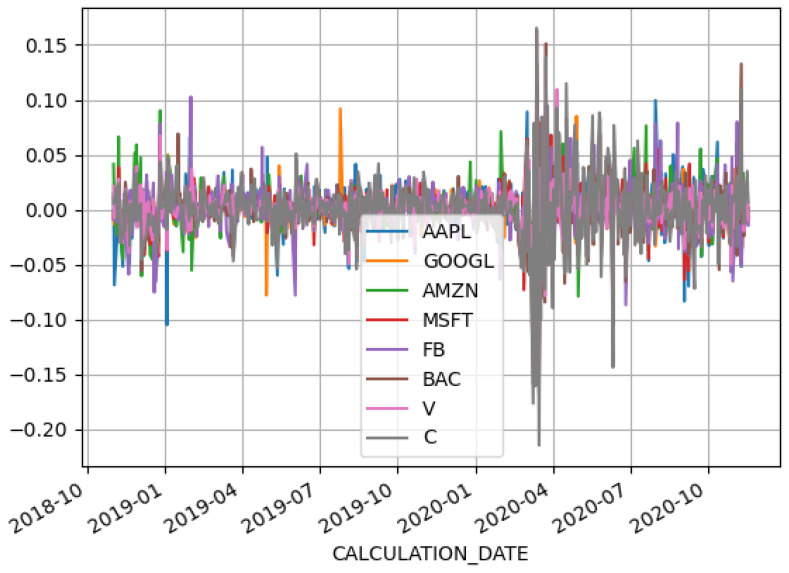
Time series plot of subset of price variables.

**Figure A4:**
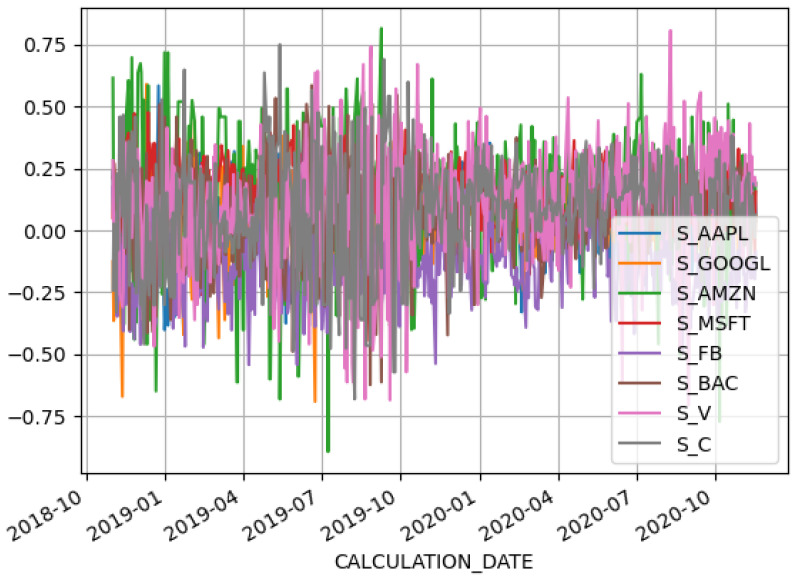
Time series plot of subset of sentiment variables.

## Data Availability

Restrictions apply to the availability of these data. Data were obtained from Brain and are available from the authors (https://braincompany.co/, accessed on 15 May 2021) with the permission of Brain.

## Appendix A

**Table A1:** Aggregated network for the following influencing sectors: Tech, Communications, Consumer Discretionary, and Consumer Staples.

Source	Target	P→P	S→S	S→P	P →S
Tech	Consumer staples	0.72	0.18	0.39	0
Tech	Healthcare	0.54	0	0	0
Tech	Financial	0.54	0	0	0.19
Tech	Industrial	0.37	0.20	0	0
Tech	Energy	0.18	0.18	0	0
Tech	Tech	0.18	0.20	0.92	0.18
Tech	Consumer discretionary	0	0.19	0	0
Tech	Communications	0	0	0	0
Communications	Healthcare	0.19	0.20	0	0
Communications	Industrial	0.18	0	0	0
Communications	Tech	0	0	0	0.20
Communications	Consumer staples	0	0.19	0	0
Communications	Communications	0	0	0	0
Communications	Consumer discretionary	0	0	0	0
Communications	Financial	0	0	0	0
Communications	Energy	0	0	0	0
Consumer discretionary	Tech	0.37	0.37	0	0
Consumer discretionary	Consumer discretionary	0.20	0	0	0
Consumer discretionary	Financial	0.19	0.19	0	0
Consumer discretionary	Industrial	0.18	0.20	0.19	0
Consumer discretionary	Consumer staples	0	0.18	0	0
Consumer discretionary	Communications	0	0	0	0
Consumer discretionary	Healthcare	0	0	0	0
Consumer discretionary	Energy	0	0	0	0
Consumer staples	Healthcare	0.19	0	0	0
Consumer staples	Tech	0.19	0.16	0	0
Consumer staples	Communications	0	0	0.20	0
Consumer staples	Consumer discretionary	0	0.18	0	0
Consumer staples	Consumer staples	0	0	0	0
Consumer staples	Financial	0	0	0	0
Consumer staples	Industrial	0	0	0	0
Consumer staples	Energy	0	0	0	0

**Table A2:** Aggregated network for the following influencing sectors: Financial, Healthcare, Industrial, and Energy.

Source	Target	P→P	S→S	S→P	P →S
Financial	Energy	0.56	0	0.17	0
Financial	Tech	0.19	0	0	0
Financial	Consumer discretionary	0.18	0	0	0
Financial	Healthcare	0.18	0	0.36	0
Financial	Consumer staples	0	0	0	0.18
Financial	Communications	0	0	0	0
Financial	Financial	0	0	0	0
Financial	Industrial	0	0	0	0
Healthcare	Energy	0.92	0	0	0
Healthcare	Tech	0.36	0.55	0	0.37
Healthcare	Industrial	0.19	0.38	0	0
Healthcare	Healthcare	0.18	0	0	0
Healthcare	Consumer discretionary	0	0.56	0.37	0
Healthcare	Consumer staples	0	0.36	0	0.18
Healthcare	Communications	0	0	0	0.55
Healthcare	Financial	0	0	0	0.20
Industrial	Tech	0.39	0	0	0
Industrial	Energy	0.20	0	0	0
Industrial	Industrial	0	0	0	0.19
Industrial	Healthcare	0	0.16	0.18	0
Industrial	Communications	0	0	0	0
Industrial	Consumer discretionary	0	0	0	0
Industrial	Consumer staples	0	0	0	0
Industrial	Financial	0	0	0	0
Energy	Tech	0.19	0	0.17	0
Energy	Communications	0	0	0.19	0
Energy	Consumer staples	0	0.18	0	0
Energy	Consumer discretionary	0	0	0	0
Energy	Financial	0	0	0	0
Energy	Healthcare	0	0	0	0
Energy	Industrial	0	0	0	0
Energy	Energy	0	0	0	0
